# Case report: A novel *HNF1A* variant linked to gestational diabetes, congenital hyperinsulinism, and diazoxide hypersensitivity

**DOI:** 10.3389/fendo.2024.1471596

**Published:** 2024-10-03

**Authors:** Suresh Chandran, Deepti Verma, Victor Samuel Rajadurai, Fabian Yap

**Affiliations:** ^1^ Division of Medicine, KK Women's and Children's Hospital, Singapore, Singapore; ^2^ Lee Kong Chian School of Medicine, Nanyang Technological University, Singapore, Singapore; ^3^ Pediatric Academic Clinical Programme, Duke-NUS Medical School, Singapore, Singapore; ^4^ Pediatric Academic Clinical Programme, Yong Loo Lin School of Medicine, Singapore, Singapore

**Keywords:** gestational diabetes, infant of diabetic mother, HNF1A variant, hypoglycemia, congenital hyperinsulinism, diazoxide hypersensitivity

## Abstract

Diazoxide (DZX) remains the first-line medication for the treatment of prolonged and persistent forms of hyperinsulinemic hypoglycemia (HH). In nearly 40%–50% of cases of HH, the genetic mechanism is unidentified. Almost half of the infants with permanent or genetic causes are DZX sensitive, but hypersensitivity to DZX is extremely rare, and the mechanism is poorly understood. Here, we report for the first time a case of DZX hypersensitivity in a neonate with HH who inherited a novel *HNF1A* variant from the mother. A term, male large-for-gestational-age infant of a diabetic mother presented with early onset of severe, recurrent hypoglycemia. Critical blood samples when hypoglycemic confirmed HH. Diazoxide was initiated at conventional doses of 5 mg/kg/day, which resulted in hyperglycemia (blood glucose, 16.6 mmol/L) within 48 h. Glucose infusion was rapidly weaned off. DZX was withheld and eventually stopped. Following 3 days of milk feeds alone with a normal glucose profile, suspecting a resolution of HH, he underwent a 6-h fasting study and passed. While on glucose monitoring in the hospital, he again developed hypoglycemic episodes, and the critical blood samples confirmed HH. DZX was restarted at a lower dose of 3 mg/kg/day, which required further down-titration to 0.7 mg/kg/day before steady euglycemia was obtained. No more episodes of hypo- or hyperglycemia occurred, and he passed a safety fasting study before discharge. Molecular genetic testing identified a novel *HNF1A* mutation in the mother–child dyad, whereas the father tested negative. We concluded that the HH phenotype due to this novel *HNF1A* mutation can be mutation specific and require a very low dose of DZX. Clinicians should observe closely for the risk of diabetic ketoacidosis and hyperglycemic hyperosmolar state while initiating DZX therapy.

## Introduction

Transient or prolonged hypoglycemia may occur in high-risk newborn infants due to impaired metabolic transition from fetal to neonatal life. Navigating the glucose nadir successfully in the first 2–3 h of life fundamentally depends on the availability of substrate and intact neuroendocrine systems to generate glucose (1). Additionally, adequate hepatic glycogen stores with unimpaired enzyme systems for glycogenolysis, gluconeogenesis, and appropriate β-cell and counter-regulatory hormone response are essential for maintaining euglycemia (2). Infants of diabetic mothers (IDMs), infants of obese mothers (IOMs), small-for-gestational-age (SGA), large-for-gestational-age (LGA), and preterm infants are prone to hypoglycemia unless they can have access to early milk feeds and, where necessary, buccal glucose gel with supplementary feeds to correct the borderline low glucose levels (3). Some infants may also require short-term parenteral glucose. Inappropriate insulin response leads to hyperinsulinemic hypoglycemia (HH), characterized by detectable insulin levels with hypoglycemia, hypoketonemia, and hypofattyacidemia. HH more often develops in IDM, IOM, SGA, LGA, and infants who had perinatal asphyxia, and the resultant neuroglycopenia enhances cerebral injury risk (4, 5).

Molecular defects in *ABCC8*, *KCNJ11*, *GLUD1*, *GCK*, *HADH1*, *SLC16A1*, *UCP2*, *HNF4A*, *HNF1A*, *HK1*, *PGM1*, *FOX A2*, *CACNA1D*, *EIF2S3*, and *PMM2* genes have been reported in patients with congenital HH. In particular, mutations in *ABCC8* and *KCNJ11* genes encoding the pancreatic K_ATP_ channel proteins, sulfonylurea receptor (SUR1), and potassium inward rectifier (Kir6.2), respectively, cause almost 50% of the genetic forms of HH. As a K_ATP_ channel agonist, diazoxide (DZX) is a first-line drug approved by the FDA (USA) for the medical treatment of HH. Consequently, genetic evaluation is warranted in HH patients who are DZX unresponsive, as mutations in K_ATP_ channel-regulating genes are more common (6–8).

In contrast to HH, maturity-onset diabetes of the young (MODY) is an autosomal dominant disease characterized by the onset of hyperglycemia before 25 years of age. There are at least 14 known MODY mutations, five of which involve the HH genes *HNF1A*, *HNF4A*, *GCK*, *ABCC8*, and *KCNJ11*. Of these, *HNF1A* and *HNF4A* encode hepatocyte nuclear factor 1α (*HNF1α*) and 4α (*HNF4α*), respectively, both of which are transcription factors for nuclear hormone receptors that are expressed in the β cells of the pancreas, thereby influencing the regulation of glucose-mediated insulin secretion (9). Therefore, mutations in these transcription factors regulating β-cell differentiation and function can cause both HH and MODY diabetes (9, 10). Heterozygous inactivating mutations in *HNF1A* and *HNF4A* genes can have a bi-phasic presentation, with transient or prolonged HH in infancy and diabetes in adolescence or young adulthood (10, 11). Additionally, we have shown that a single *HNF4A* mutation can cause variable phenotypes among members of the same family (12). HH due to *HNF1A*, and *HNF4A* gene mutations respond well to DZX. Interestingly, Arya et al. reported exceptional diazoxide sensitivity to a conventional dose of DZX in an HH infant with a novel *HNF4A* mutation requiring lower doses of DZX for glucose control (13). Here, we describe an HH infant with a novel maternally inherited *HNF1A* mutation presenting with an ultra-responsiveness to a traditional dose of DZX.

## Case presentation

A term, male infant was born at 38 + 3 weeks via cesarean section for cephalopelvic disproportion to a non-consanguineous Chinese couple. The mother had gestational diabetes on diet control (HbA1c 8.4%, 32 weeks gestation). The baby was non-dysmorphic, LGA (4,280 g, 98th percentile, +2.16 SDS), had an occipitofrontal circumference of 34 cm (45th percentile, −0.13 SDS), and had length of 56 cm (99th percentile, +2.71 SDS). There was a family history of type 2 diabetes and obesity in the maternal grandfather and type 2 diabetes in the grandmother.

At the hospital of birth, the infant had symptomatic hypoglycemia at 2 h of life, with jitteriness, and the capillary blood glucose (CBG) was 0.7 mmol/L. Hypoglycemia was refractory to a mini-bolus of 10% dextrose, requiring a progressive escalation of glucose infusion rate (GIR) to a maximum of 22.4 mg/kg/min to achieve euglycemia. Hence, he was transferred to our tertiary neonatal center on day 7 of life and underwent a controlled GIR reduction test to obtain the critical diagnostic blood, which confirmed HH (plasma glucose, 2.1 mmol/L; insulin, 42.3 mIU/L; ketone, 0.1 mmol/L; and free fatty acids, 0.12 mmol/L). Growth hormone and cortisol response to hypoglycemia were appropriate. Blood gas, renal and liver function tests, an inborn error of metabolism screen, acylcarnitine, and infective markers were unremarkable. An echocardiogram prior to initiation of DZX revealed asymmetric septal hypertrophy.

For the symptomatic hypoglycemia and high GIR requirements, he was initiated on a standard oral dose of DZX, 5 mg/kg/day in three divided doses on day 8 of life, and hydrochlorothiazide was added. Over 48 h, he responded to DZX with a glucose profile in the 3.5–8 mmol/L range. On day 10, CBG peaked at 10 mmol/L, and on day 11, while weaning of GIR was in progress, his CBG levels reached 16.6 mmol/L, indicating a possible spontaneous resolution of HH. DZX was withheld, and glucose infusion was progressively weaned and ceased. He was kept on close CBG monitoring while continuing on milk feeds, and the glucose profile remained stable for the following 72 h. To confirm a resolution of HH, a 6-h fasting study was conducted on day 15 of life, which he passed.

**Figure 1:**
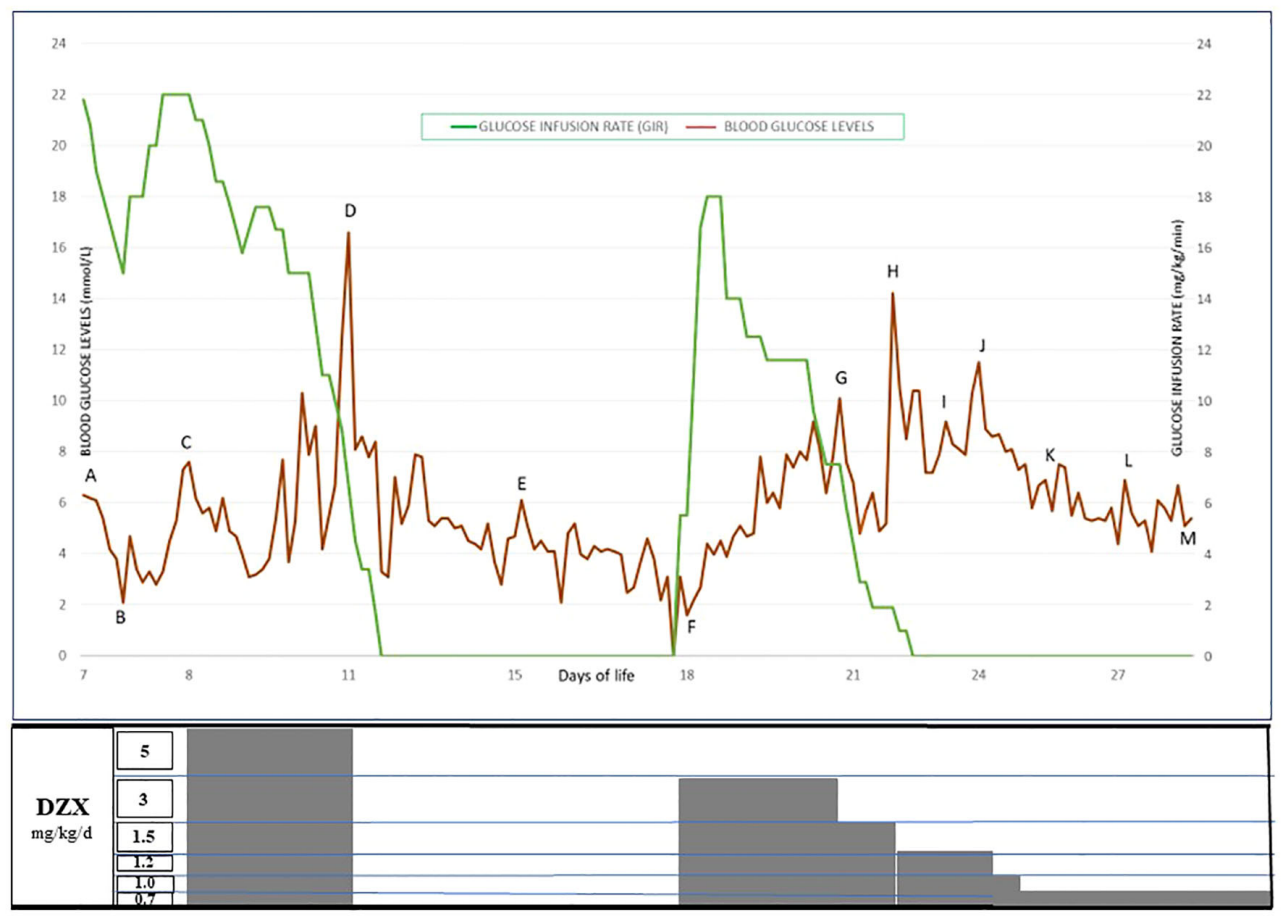
Timeline of events from day 7 to day 28 of life. Glucose profile and glucose infusion rates are depicted. Diazoxide dose given is shown in the box below. **(A)** Controlled GIR reduction test to confirm HH; **(B)** confirmation of HH; **(C)** initiation of DZX at 5 mg/kg/day; **(D)** DZX discontinuation; **(E)** passed fasting study; **(F)** hypoglycemia, reinstating glucose infusion followed by DZX at 3mg/kg/day; **(G–I)** DZX dose down-titrated to 1.5 mg/kg/day, 1.2 mg/kg/day, and 1 mg/kg/day; **(J)** omitted a dose of DZX; **(K)** DZX dose reduced to 0.7 mg/kg/day; **(L)** passed safety fasting study; **(M)** discharged home. DZX, diazoxide; GIR, glucose infusion rate; HH, hyperinsulinemic hypoglycemia.

**Figure 2:**
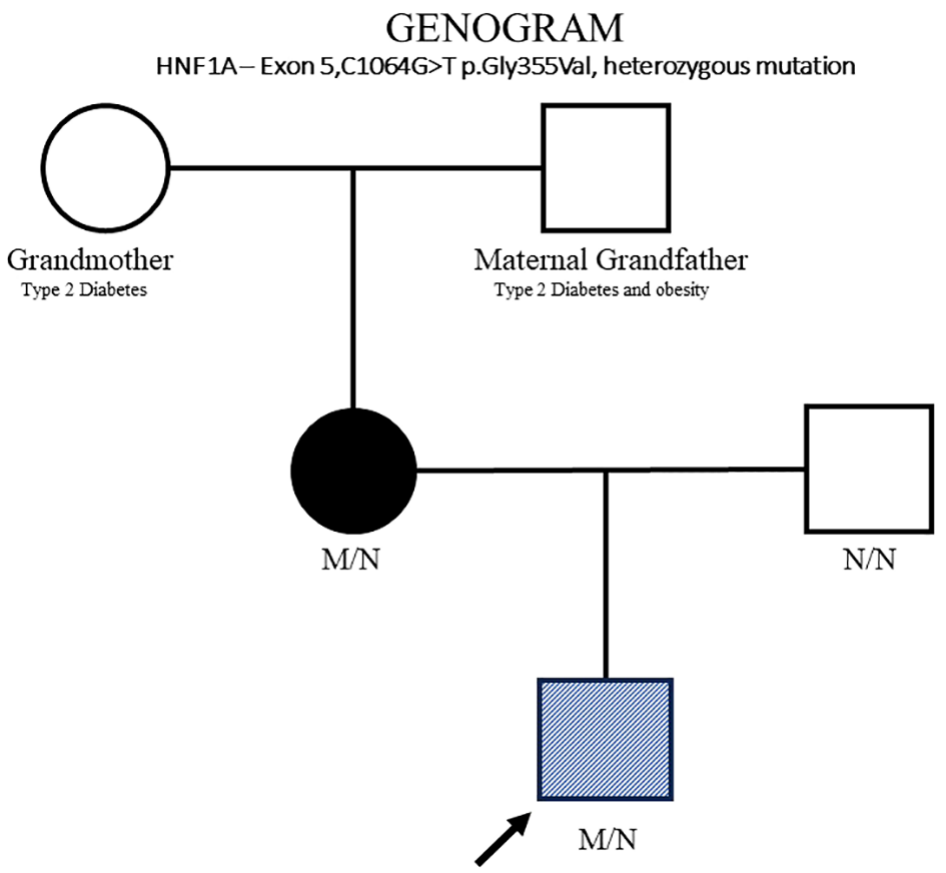
HNF1A family pedigree. Circles (female) and squares (male). Diagonal hatching denotes HH (proband). Filled symbols indicates MODY (mother). Unfilled symbol indicates normal genotype (father) and maternal grandfather and grandmother. M/N, heterozygous *HNF1A* genotype; N/N, normal genotype.

On day 16 of life, the infant again developed hypoglycemia while on enteral nutrition, which spontaneously resolved with feeds. Recurrent hypoglycemic episodes were noted again on days 17–18 of life, even to unrecordable glucose levels. Glucose infusion was restarted, and the maximal GIR required was 18 mg/kg/min. Critical blood samples (plasma glucose, 1.5 mmol/L; insulin, 26.2 mIU/L; ketone, 0.0 mmol/L; and free fatty acids, 0.18 mmol/L) again confirmed HH. Thus, a lower dose of DZX, 3 mg/kg/day, was initiated on day 18 of life. Again, a rise in CBG levels up to 14 mmol/L necessitated further down-titration of DZX, and the CBG levels became stable on a dose of 0.7 mg/kg/day of DZX (**Figure 1**). On this low dose of DZX, he passed a 6-h safety fasting study before discharge to ensure the maintenance of CBG levels during an inadvertent fast at home. Parents underwent counseling and caregiver training with an action plan for hyperglycemia and hypoglycemia. Genetic testing was done using the Invitae (San Francisco, CA, USA) hypoglycemia panel (120 genes). A novel heterozygous mutation in exon 5,c.1064G>T(p.Gly355Val) was identified, replacing glycine with valine at codon 355 of the *HNF1A* protein. Further family genetic testing revealed the same genetic mutation in the mother, and the father tested negative (**Figure 2**).

## Outcome and follow-up

He remained on home glucose monitoring, and the CBG chart was reviewed online by the hypoglycemia nurse on a day-to-day basis and weekly by a team consultant. Till 5 months of age, his CBG remained stable. Then, occasional nocturnal glucose levels became borderline low, and the dose of DZX was raised from 0.7 mg/kg/day to 1 mg/kg/day, implying the persistence of HH. He required a further stepwise increase in the DZX dose over the next 5 months to 2.5 mg/kg/day, as borderline low glucose levels were recorded at night while on formula milk, corn starch, and weaning foods under the dietitian’s advice. Neurodevelopmental assessment and growth parameters, namely, weight of 11.5 kg (95th percentile, +1.62 SDS), occipitofrontal circumference of 48 cm (93rd percentile, +1.51 SDS), and length of 81 cm (99th percentile, +2.21 SDS) were all above the 90th percentile at 12 months of age.

## Discussion

We report a term LGA infant of a diabetic mother with congenital hyperinsulinism who demonstrated exquisite sensitivity to 3–5mg/kg/day of DZX, with CBG reaching the diabetic range. Hyperinsulinism was not transient but recurred, and the baby required a very low dose of DZX <1 mg/kg/day to maintain euglycemia. The very low dose requirement was transient such that by 5 months old, a higher but lower than standard dosing of DZX was needed to maintain his normal glucose profile. The baby had a unique *HNF1A* variant, and we postulate that this may be responsible for the changing requirements in the first year of life.

Arya et al. reported a neonate with exceptional DZX sensitivity with a dose of 5 mg/kg/day in the background of a novel *HNF4A* mutation in the mother–child dyad. Later, a reduced dose of DZX, 1.5mg/kg/day, stabilized the CBG levels (13). To the best of our knowledge, there is no report of DZX hypersensitivity needing an extremely low dose of DZX (0.7 mg/kg/day) to maintain euglycemia in a confirmed case of HH having an *HNF1A* mutation. *HNF1A* mutation is novel in our case, and the predisposition to DZX hypersensitivity could be mutation specific.

MODY accounts for at least 1% of all cases of diabetes mellitus (14). In the UK, 80% of MODY variants were misdiagnosed as T1/T2 diabetes (15). With increasing awareness of MODY, more novel mutations are identified using genetic testing and reported with diabetes in pregnancy and adulthood and HH in infants. Pearson et al. and Fajan et al. identified *HNF4A* mutations causing a biphasic phenotype, HH, and MODY1 (16, 17). Earlier reports by Pearson et al. did not find a higher prevalence of HH in *HNF1A* variants. But later, Tung et al. identified 12 cases of diazoxide-responsive HH in the USA, of which 58% had *HNF1A* mutation, and Rozenkova et al. reported that *HNF1A* mutations represented the second most frequent cause of HH in the Czech Republic (18, 19).

Our described neonate was LGA and had symptomatic hypoglycemia within hours of birth. Heterozygous mutations in *HNF4A* were known to cause macrosomia but not with *HNF1A* (16). However, two recent reports point to a fetal overgrowth *in utero* and neonatal HH with *HNF1A* mutation (20, 21), as noted in our case. Most cases of HH in IDM are transient and resolve within several days to a week (22). The hypoglycemia in our infant was due to hyperinsulinism. Even though HH was hypersensitive to DZX initially, the response to treatment was appropriate for HH while on a very low dose. The course of HH continues beyond infancy, and the requirement of DZX dose rose with age, supporting the nature of some of the MODY gene mutations (12, 13).

Determining whether the given novel *HNF1A* variant of uncertain significance (VUS) is responsible for HH is challenging. Still, GDM, fetal overgrowth, and persistent neonatal HH with the same novel mutation in the mother–child dyad suggest a MODY phenotypic presentation. Cromer et al. and Yau et al. reported a similar scenario to our case, where a single genetic VUS in *HNF1A* caused gestational diabetes, fetal overgrowth, and neonatal HH (11, 20). The mutation identified in our case is currently a VUS, as this variant is not present in population databases (Exome Aggregation Consortium). The identified mutation was in exon 5,c.1064G>T (p.Gly355Val), replacing glycine with valine at codon 355 of the *HNF1A* protein. The glycine residue is moderately conserved, and there is a moderate physicochemical difference between glycine and valine. Further advanced modeling of protein sequence and biophysical properties performed at Invitae laboratory (structural, functional, and spatial information, amino acid conservation, physicochemical variation, residue mobility, and thermodynamic stability) indicates that this missense variant is expected to disrupt *HNF1A* protein function. This observation further confirms that the location or characteristics of the specific genetic variant can derange the protein function or degree of penetrance and can present with different phenotypes. Given the absence of population data and lack of functional studies on protein function, available evidence is currently insufficient to determine the role of this variant in disease.

Successful use of DZX to treat hypoglycemia began in 1960 (23). The recommended dose of DZX to treat HH is 5–20 mg/kg/day. A history of non-ketotic hyperosmolar coma with DZX therapy was first reported in a 13-month-old boy with HH by Balsam et al. The infant was on 7.5 mg/kg/day of DZX and developed listlessness and seizures within a week of initiation of DZX. The plasma glucose was noted to be 2,000 mg/dL (111.1 mmol/L). On recovery from hyperglycemia, he was restarted on a lower dose of DZX at 4 mg/kg/day and maintained optimal glucose control. No genetic testing was done in this case (24). In 2018, Mangala et al. reported a 16-month-old with hyperglycemic (>22 mmol/L) hyperosmolar coma and ketoacidosis while on 15 mg/kg/day of DZX for HH, diagnosed at 4 months of age. No pathogenic variant was detected in genetic testing (25). In both cases, hyperglycemia occurred during intercurrent illnesses, and the dose of DZX used was much higher than in our case. Diazoxide is metabolized in the liver and excreted through the kidneys. While on treatment with DZX, intercurrent illnesses could impair hepatic and renal functions, and the DZX levels could rise exponentially to toxic levels manifesting as hyperglycemia and hyperosmolar coma. There was no record of hepatic function in the former, but in the latter, they reported marked hepatic and renal dysfunction, which could account for the DZX toxicity (24, 25). In our reported case, he developed hyperglycemia initially while on a recommended lower end of conventional DZX dose. He was screened negative for infections. Hyperosmolar ketosis was prevented in the index case because of close monitoring and timely intervention. Recently, there were reports of increasing adverse reactions to DZX in newborn infants (26). Two recent publications have shown the safety and efficacy of low-dose DZX in treating HH in SGA, non-SGA, preterm, and term infants (27, 28).

The mechanisms underlying the hyperresponsiveness to DZX are unknown. Higher blood glucose levels while on DZX treatment could involve a mechanism similar to the dual phenotypic presentation in the *HNF1A/HNF4A* mutations, switching from hypoglycemia to hyperglycemia. Stanescu et al. suggested a possible relationship between *HNF1A*, *HNF4A*, *GLUT2*, and the K_ATP_ channel. She described two *HNF1A* children with congenital hyperinsulinism, who, unlike ours, had variants that were paternally inherited, and neither child demonstrated diazoxide hypersensitivity. A potential mechanism for diazoxide hypersensitivity involves interaction between the transcription factors *HNF1A* and *HNF4A*, and the K_ATP_ channel (10). It is presently unclear whether the inheritance pattern may play a role.

This case study identified a strong family history of diabetes in the grandparents of the index case, but due to old-age illnesses, no genetic testing was done. MODY is an autosomal dominantly inherited disease, with gestational diabetes in the mother who has the same novel mutation as the proband. It is plausible that the maternal grandparents, too, have the same mutation. Long-term management of infants with an *HNF1A* variant includes understanding its triphasic nature and the transitions from hypoglycemia to normoglycemia to diabetes. Genetic counseling for future pregnancies will need to include highlighting the autosomal dominant nature of this variant, and the risks of diabetes in pregnancy, neonatal hypoglycemia, and early-onset hyperglycemia in individuals who have the variant.

## Strengths and limitations

As with all case reports, the key limitation lies in its generalizability, except that this is the second report of a hepatocyte nuclear factor variant linked with exquisite DZX sensitivity.

## Conclusion

Like hypoglycemia, DZX itself is not benign. This case study implies that a very low dose of DZX may be sufficient in infants with this reported novel *HNF1A* variant. We postulate that abnormalities in genes encoding transcription factors may alter the affected infant’s sensitivity to DZX. It is justifiable to do a genetic study in prolonged and persistent HH in IDM, especially in the background of a strong family history of early onset diabetes. Parents should be educated on the possibility of hyperglycemia and hypoglycemia while on DZX treatment to prevent hyperglycemia and hyperosmolar coma to avoid neuronal injury.

## Data Availability

The original contributions presented in the study are included in the article/supplementary material. Further inquiries can be directed to the corresponding authors.
